# Ion channels and neuropathic pain

**DOI:** 10.7554/eLife.42849

**Published:** 2018-11-26

**Authors:** Madeleine C Klein, Anne Louise Oaklander

**Affiliations:** 1 Center for Genomic Medicine, Department of NeurologyMassachusetts General HospitalBostonUnited States; 2 Harvard Medical SchoolHarvard UniversityBostonUnited States; 3 Department of NeurologyMassachusetts General HospitalBostonUnited States; 4 Department of PathologyMassachusetts General HospitalBostonUnited States

**Keywords:** Fabry disease, small fibers, neuropathy, Gb3, α-GAL, Mouse

## Abstract

Pain behaviors in a Fabry mouse model are associated with the accumulation of a fat molecule that disrupts sodium ion channels in small fiber neurons.

**Related research article** Hofmann L, Hose D, Grießhammer A, Blum R, Döring F, Dib-Hajj S, Waxman S, Sommer C, Wischmeyer E, Üçeyler N. 2018. Characterization of small fiber pathology in a mouse model of Fabry disease. *eLife* **7**:e39300. doi: 10.7554/eLife.39300

In our bodies, peripheral nerve cells of different thicknesses connect the brain to the outside world. Most of these neurons are unidirectional. They either send information to our brain about what takes place in or around the body, or they respond and then relay signals outward from the brain to the muscles and other cells. However, least-evolved neurons called C-fibers still work in ancient and undisciplined ways, and do not always obey the rules of neuroanatomy. For instance, they are bidirectional: in addition to encoding and transmitting messages inward to the spinal cord and the brain when they encounter dangerous stimuli, these small diameter neurons also convey signals outward to a wide range of nearby cells throughout the body (Fukuda et al., 2013; Dori et al., 2015).

It is no wonder, then, that peripheral neuropathies which damage small fibers lead to a bewildering array of symptoms: chronic widespread pain that often starts in the feet and legs, dizziness, weakness with exertion (also known as chronic fatigue), fainting upon standing, nausea, constipation or diarrhea, and itching (Terkelsen et al., 2017). With such nonspecific symptoms, it is often difficult to diagnose the neuropathy and then its underlying medical cause. Although neuropathy often appears because of diabetes or toxic exposures, in very rare cases it can emerge because of genetic mutations. Studying these exceptional patients is a time-honored way of figuring out the mechanisms that lead to these symptoms.

One such example is Fabry disease, a rare inherited disorder caused by mutations on the X-chromosome, meaning it affects males more often and more seriously than females. Children with the condition often begin to notice episodes of burning pain, less sweating in their feet and hands, and digestive difficulties. This pain can flare up when their temperature rises as a result of exercise, fever or hot weather. The mutation responsible for Fabry disease targets an enzyme called α-22 galactosidase A (α-GAL), an enzyme that breaks down and helps recycle a fat molecule known as Gb3 (short for globotriaosylceramide). When the enzyme is not fully functional, Gb3 is not degraded and instead accumulates inside the cell.

The α-GAL enzyme is expressed in small fiber neurons and the lining of blood vessels. Because small fibers also partly regulate many blood vessels, dysfunctional α-GAL is a double blow to the circulatory system. Indeed, when patients get older, many develop vascular problems such as strokes, and heart and kidney damage (Gupta et al., 2005). As treatments that aimed to replace α-GAL were developed, some countries and states began to screen newborns for Fabry (Hopkin et al., 2016). However, researchers did not fully understand how the accumulation of undigested Gb3 caused small fiber neurons to fail.

Now, in eLife, Nurcan Üçeyler of the University of Würzburg and co-workers at Würzburg and Yale Medical School and Veterans Affairs Hospital – including Lukas Hofmann as first author – fill in some of the blanks (Hofmann et al., 2018). The team elegantly combined molecular, histological, electrophysiological and behavioral techniques to study a mouse model of Fabry disease in which the gene for α-GAL has been deactivated (or ‘knocked out’). It was already known that as these mice get older, Gb3 accumulates within and around the cell bodies of their small fiber neurons in the sensory ganglia (Gadoth and Sandbank, 1983; Hofmann et al., 2018). Located near the spinal cord, these ganglia help the body process sensations.

Hofmann et al. went on to examine how the α-GAL knockout mice respond to pain – for example, how long it takes them to withdraw a hind paw from a hot surface. When comparing young and old animals with or without the mutation, the team showed that older mice with the genetic change developed abnormal pain responses. In these animals, the farthest ends of the small fiber neurons had degenerated, which is the pathological hallmark of small fiber neuropathy.

The team then focused on three specific ion channels that are important to small fiber function, recording their activity using a method known as patch clamp. Ion channels are proteins which span the cellular membrane and open or close in response to the local environment. This allows ions to pass into and out of the cell to create an electrical signal that can travel along the neuron, making it ‘fire’. In older mice, the function of two of these ion channels, HCN2 and the sodium channel Na_V_1.7, had deteriorated as compared to normal controls. Not surprisingly, both channels had already been linked to small fiber neuropathic pain (Emery et al., 2011; Faber et al., 2012).

Then, as a coup de grace, Hofmann et al. used RNA interference to silence α-GAL in a culture of embryonic kidney cells that expressed Na_V_1.7 channels on their membranes. This caused Gb3 to accumulate in these cells, and their sodium currents to falter. When the cells were then exposed to an existing treatment for Fabry disease, which restores α-GAL, healthy Na_V_1.7 currents were reestablished and the Gb3 deposits decreased.

Although the work by Hofmann et al. does not explain how the accumulation of Gb3 affects Nav1.7 channels, or clarify if other ion channels are involved, it does demonstrate that increased amounts of Gb3 can lead to pathologic, physiologic, and behavioral signs of neuropathy. Armed with this knowledge, researchers might be able to develop new, non-genetic ways to reduce Gb3 deposition. This could be valuable to Fabry patients, as the current α-GAL replacement treatment is prohibitively expensive and does not always ensure that Gb3 is broken down around the clock. The cell lines and methods developed by Hofmann et al. could also help researchers study other disorders where waste products are not properly recycled and to identify other, more common, causes of small fiber neuropathy.
